# Coordinated ramp signal optimization framework based on time series flux-correlation analysis

**DOI:** 10.7717/peerj-cs.446

**Published:** 2021-03-25

**Authors:** Zhi Liu, Wendi Shu, Guojiang Shen, Xiangjie Kong

**Affiliations:** College of Computer Science and Technology, Zhejiang University of Technology, Hangzhou, Zhejiang, China

**Keywords:** Ramp signal optimization, Correlation analysis, GRU neural network

## Abstract

Urban expressways provide an effective solution to traffic congestion, and ramp signal optimization can ensure the efficiency of expressway traffic. The existing methods are mainly based on the static spatial distance between mainline and ramp to achieve multi-ramp coordinated signal optimization, which lacks the consideration of the dynamic traffic flow and lead to the long time-lag, thus affecting the efficiency. This article develops a coordinated ramp signal optimization framework based on mainline traffic states. The main contribution was traffic flow-series flux-correlation analysis based on cross-correlation, and development of a novel multifactorial matric that combines flow-correlation to assign the excess demand for mainline traffic. Besides, we used the GRU neural network for traffic flow prediction to ensure real-time optimization. To obtain a more accurate correlation between ramps and congested sections, we used gray correlation analysis to determine the percentage of each factor. We used the Simulation of Urban Mobility simulation platform to evaluate the performance of the proposed method under different traffic demand conditions, and the experimental results show that the proposed method can reduce the density of mainline bottlenecks and improve the efficiency of mainline traffic.

## Introduction

Traffic congestion on urban roads is an important issue that needs to be addressed in smart cities’ development. Furthermore, in addition to road congestion in the city center, urban expressways, as the hub connecting work areas and living areas, have a high load concentration during commuting, causing extensive congestion and spread quickly.

The most direct and convenient way to address high traffic congestion is ramp signal duration adjustment (Papageorgiou & Kotsialos, 2002). While the expressway suffers heavy congestion during the morning and evening rush hours, the adjacent ramps are under tremendous traffic pressure. The local ramp signal optimization only considers the traffic status of one section of the ramp convergence area of the central expressway, which may aggravate the overall traffic congestion and may reduce the effective utilization of upstream-downstream traffic facilities (Zhang & Wang, 2013). When there are multiple bottlenecks on the mainline or the limited capacity on the ramp, coordinated ramp signal optimization that aims at stabilizing the smooth flow on the mainline and prevent queue spillback on multiple ramps may be more effective than local optimization. In recent years, as the sensor is all over a wider range, and the accuracy is improved, real-time monitoring of urban traffic is achievable—the analysis of big data and the processing of data-driven methods to optimize the traffic signal become novel solutions (Feng et al., 2019).

Existing methods use only local information, such as spatial distance, OD information (Chen et al., 2019b). They determine the traffic flow priority without consideration of the similarity of traffic patterns between the congested section and the on-ramp. Traffic flow is time-varying, and similar traffic flow patterns imply that the on-ramp has higher importance for congestion at that moment, and the lack of this consideration leads to a decrease in control efficiency. Besides, the traditional approach lacks consideration of traffic flow evolution trends and future traffic patterns, which can lead to a long time-lag.

In this article, we propose a coordinated ramp signal optimization framework based on time series flux-correlation analysis. Initially, we collected the historical traffic data of the road from the PeMS database. Then, we used neural networks to make online predictions about traffic flow every 3 h and measured the predicted flow-series correlation between the congested section and ramps. Furthermore, based on the gray correlation analysis, we compared the similarity of the three traffic factors’ curves, including the correlations, spatial distance and traffic volume, with the curves of speed and obtain the corresponding contribution weights of each attribute. Finally, we used a heuristic strategy to optimize multiple-ramps signals with competing priorities coordinately. We compared the performance of the proposed method and the traditional method under different traffic demands. Our contributions are summarized as follows:

- *Traffic flow prediction*. The traffic data of the congested road and ramps are predicted by GRU neural network in the time dimension to obtain the traffic flow at the future time to ensure real-time and prospective optimization.
- *Flux-correlation measurement*. The correlation of the traffic flow-series between the congested section and the upstream ramps is obtained by the cross-correlation method.
- *Novel metrics for flow priority*. Together with distance and predicted on-ramp flow, the correlations establish the weight matrix, allocating the excess traffic demand.
- *Heuristic ramp signal optimization*. The bottleneck algorithm is used to implement our signal optimization framework based on the weight matrix.

The remainder of the article organized as follows: “Literature Review” describes the traffic flow prediction method and the time-series correlation measurement; “Framework Design” demonstrates how the coordinated ramp signal optimization framework developed; “Experiment” evaluates the performance of the method based on the Simulation of Urban Mobility (SUMO) simulation platform and “Results” summarizes the full text.

## Literature review

### Traffic flow prediction

Using large-scale historical traffic data for traffic flow prediction and solving traffic management, guidance and route planning problems have become a hot research topic today (Uras et al., 2020). In essence, traffic flow forecasting is the extraction of experience and knowledge from relevant historical data to estimate the future state. Previous studies can usually be divided into parametric and nonparametric methods (Zhang et al., 2018). Parametric methods provide simple estimates of future traffic conditions with low computational complexity. However, they are only applicable to specific traffic data conditions because changes in external conditions and the randomness and nonlinearity of traffic flow can impact prediction accuracy (Kong et al., 2020, 2021).

Time series analysis models use curve fitting and parameter estimation to predict future traffic flow information. The most typical method is the autoregressive integrated moving average (ARIMA). The ARIMA model adds an integral link to the autoregressive and sliding average models to eliminate short-term fluctuations in the time series. Many variants have been derived based on ARIMA, such as SARIMA (Williams & Hoel, 2003), which adds a periodic term to this base. This method is suitable for smoother traffic flows and is not sufficient for predicting complex traffic conditions.

Nonparametric models have unique advantages. However, these traditional machine learning methods require labeling data in model training. Furthermore, these methods capture traffic flow characteristics using artificial features, making it difficult to achieve desirable prediction results. Kumar, Parida & Katiyar (2013) used traffic volume, speed, density and time as input variables and used the artificial neural network for short time traffic flow prediction.

Many deep learning models have been proposed to solve the traffic flow prediction problem with the more expansive urban road sensing device arrangement and improved recognition accuracy. Stacked Autoencoder (SAE) (Lv et al., 2014) uses a hierarchical greedy algorithm to obtain spatio-temporal traffic flow characteristics. Recurrent neural networks have been widely used for short-time traffic flow prediction because of their ability to process arbitrary length inputs using memory units. LSTM and GRU (Cho et al., 2014) derived from RNN have shown better prediction performance. Zheng et al. (2020) proposed a convolutional LSTM neural network based on attention mechanism to extract the Spatio-temporal features of traffic flow. They also use Bi-LSTM module to extract the daily and weekly long-term features of traffic flow. Liu, Wang & Zhu (2018) used a gated CNN instead of LSTM to extract the temporal features of traffic flow and combined with CNN’s spatial features to predict the traffic flow. Besides, Traffic flow prediction with graph convolutional neural networks is becoming a trend (Shen, Zhao & Kong, 2021). Han proposed Dirgraph Convolutional Neural Network to tackle the congestion recognition problem through a new graph feature extraction method (Han et al., 2020).

In this article, we use the GRU neural network for traffic flow prediction. Firstly, we mainly forecast the downstream section’s traffic volume, so the traffic sequence’s temporal characteristic should be mainly concerned. Secondly, the traffic flow of the freeway is relatively stable. The variation of traffic flow in each section of the freeway mainly depends on the traffic flow entering and leaving the ramp, so we do not consider the traffic flow’s spatial characteristics. Finally, compared with other neural network methods, the design of GRU is more straightforward and meets our requirements for operability.

### Time-series correlation

The correlation measurement between the vectors generally achieves through the distance matrix, including Euclidean distance (Berkhin, 2006) and Manhattan distance (Bakar et al., 2006). Conventional methods are generally used for two time-series data of the same length and different time lags. However, since there is necessarily a time lag between the traffic flow time series, there is no point-to-point correspondence between the two on the time axis. Researchers have come up with several ways to overcomes this shortcoming, such as dynamic time warping (Berndt & Clifford, 1994) and cross-correlation (Liao, 2005). In this article, cross-correlation is selected for correlation measurement. It has been applied to anomaly detection of key performance indicators (Li et al., 2018) and the classification of traffic smart card data (He, Agard & Trépanier, 2020). Meanwhile, it is also widely used in the field of time series clustering (Paparrizos & Gravano, 2015).

Su et al. (2019) first proposes the concept of flux-correlation. This research proposed an unsupervised method to determine the correlation of server KPI fluctuations as well as the direction of fluctuations. If the fluctuations of one series are correlated with the fluctuations of another series over a period of time, then define two series are flux-correlated. We compare the flux-correlation between ramps and bottleneck sections in this article to explore the correlation between flow fluctuations.

### Multi-ramp coordination signal optimization

In addition to ramp signal optimization, other methods include the mainline variable speed limit. However, because there are few mainline variable speed limit applications, the actual deployment is difficult and the safety risks are high. Therefore, MPC control strategy is mostly used at present, which is a typical nonlinear optimal control method that can predict the control effect to achieve control optimality, most commonly by combining the mainline variable speed limit method and the ramp control method to achieve synergistic control of both (Hegyi, De Schutter & Hellendoorn, 2005; Van de Weg et al., 2019).

Multi-ramp coordination signal optimization methods can be mainly divided into model-based and model-free methods (Papageorgiou & Kotsialos, 2002). The optimal coordination method is realized based on the traffic flow model. According to the real-time traffic flow information, the control rate is solved by taking the shortest travel time and waiting time as the control objectives and the mainline capacity and ramp queue length as the constraints. Traffic flow models such as the Payne model (Chang & Li, 2002), the cell transmission model (Chen, Lin & Jiang, 2017; Meng & Khoo, 2010; Schmitt, Ramesh & Lygeros, 2017) and METANET (Dabiri & Kulcsar, 2017; Frejo & Camacho, 2012; Kontorinaki, Karafyllis & Papageorgiou, 2019) are widely used. Meshkat applied a quantitative hierarchical model to ramp coordination signal optimization for the first time (Meshkat et al., 2015). Chen added real-time OD information based on Meshkat to determine the priority of on-ramp flow through the total vehicle travel distance (Chen et al., 2019b).

In the field of the model-free method, most researches focus on various heuristic algorithms, such as HELPER (Lipp, Corcoran & Hickman, 1991), SWARM (Paesani et al., 1997) and HERO (Papamichail et al., 2010b). In meanwhile, many researchers adopt a data-driven approach to optimize ramp signals. Chen uses large-scale traffic data and integrates external weather factors to analyze and model the evolution pattern of traffic congestion. The signal duration adjusts dynamically through dynamic congestion threshold classification and congestion mode clustering. For the first time, the analysis of big traffic data was applied to ramp signal optimization, with more careful considerations and more consistent with the actual situation (Chen et al., 2019a). Zhang uses large-scale vehicle trajectory data to extract the vehicle on-ramp behavior pattern, trace the source of congestion formation, and optimize the signal duration of multiple ramps (Zhang et al., 2019).

We combine the dynamic traffic flow correlation based on the traditional heuristic method. The excess traffic demand is assigned by tracing the distribution weights of the congestion sources.

## Framework design

**Figure 1:**
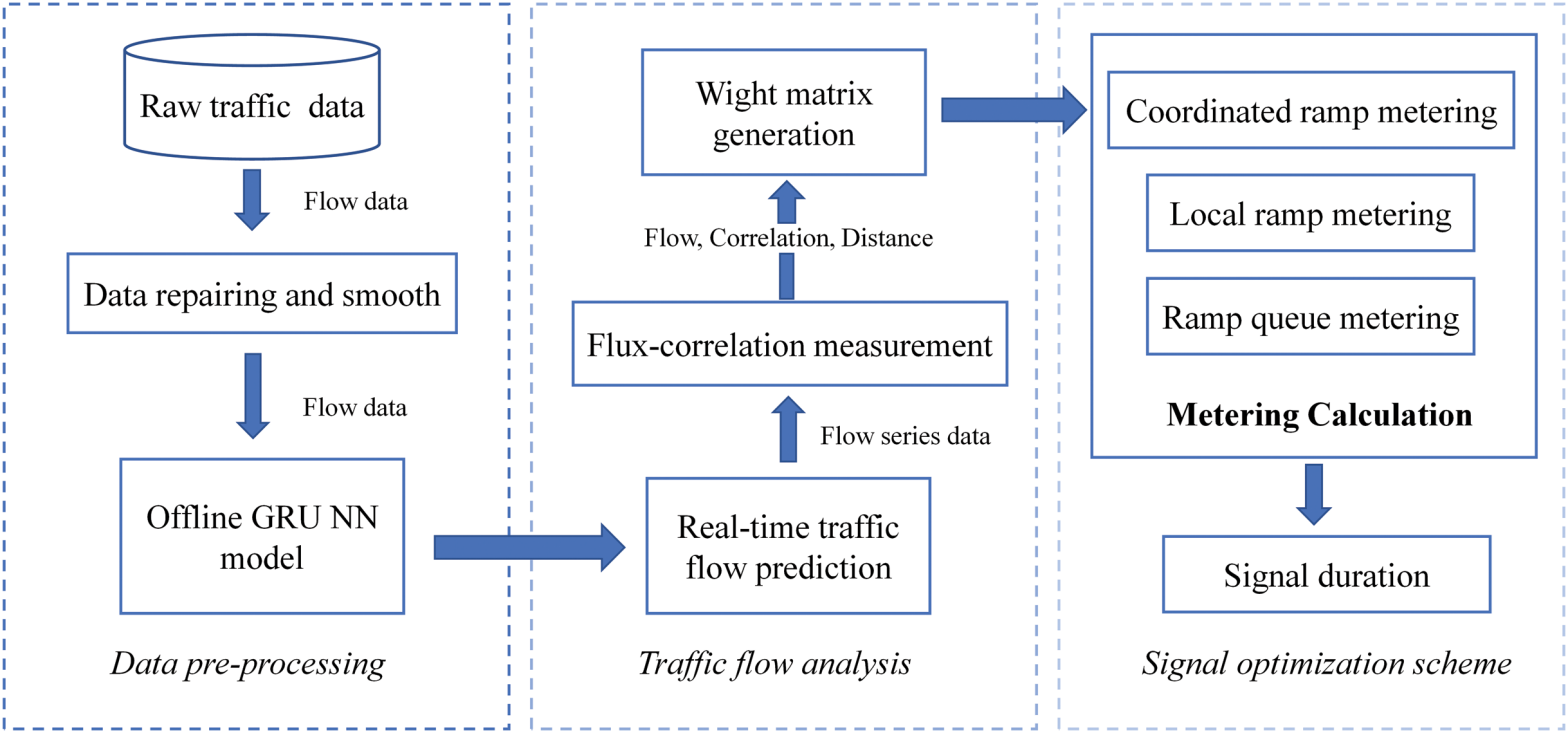
Multi-ramp coordinate signal optimization framework.

In this article, the coordinated signal optimization framework is divided into three parts: data pre-processing, traffic flow analysis and signal optimization scheme generation. The coordinated signal optimization framework aims to combine dynamic traffic flow information to optimize real-time signals dynamically. Initially, the framework first restores and organizes the raw traffic data through the data pre-processing module. Furthermore, the smoothed historical traffic flow data is trained to obtain the offline model. Secondly, we predict the real-time traffic flow data by the traffic flow analysis module and perform correlation analysis on the predicted traffic data. The weight matrix is obtained by combining distance, ramp flow and traffic correlation. Finally, we develop a coordinated signal optimization scheme for signal timing of multiple ramps. The structure of framework is shown in Fig. 1.

### Data pre-processing

Time series data is a set of observations, and time series are generally discrete collections of discrete points that contain temporal relationships in contrast to other vectors. A set of traffic flow time series data is continuous observed values collected by a loop detector according to equally spaced time stamps. A set of time series can be represented as }{}Q{\rm = }\left[ {{q_1},{q_2},\ldots,{q_i},\ldots,{q_n}} \right], where }{}{q_i} is the observed traffic flow value based on the time index value. But the raw data is not organized and needs to be filtered, repaired and smoothed. After data cleaning, it will become serial data.

The raw traffic data is the traffic information of each highway’s detection point at a certain moment. We choose roads with complete data. Make sure that there is the least amount of missing data in two consecutive month time periods. The final data for the experiment is obtained and some examples are shown in Table 1 below.

**Table 1:** An example of the processed data.

Timestamp	Station	Direction	…	Total flow (vel)	Avg occupancy (%)		Avg speed (km/h)
02/05/2019 17:15:00	763720	N	…	376		0.3254	18.9
02/05/2019 17:20:00	763720	N	…	412		0.322	19.8
02/05/2019 17:25:00	763720	N	…	314		0.4013	17.6
02/05/2019 17:30:00	763720	N	…	319		0.4011	17.7
02/05/2019 17:35:00	763720	N	…	330		0.4087	16.9
02/05/2019 17:40:00	763720	N	…	350		0.3654	17.3
02/05/2019 17:45:00	763720	N	…	362		0.3815	17.5
02/05/2019 17:50:00	763720	N	…	333		0.3778	17.5
02/05/2019 17:55:00	763720	N	…	319		0.3929	17.7
02/05/2019 18:00:00	763720	N	…	397		0.1774	24.8

Although the quality of the data obtained through screening is high, noise and missing data inevitably occur, which can negatively affect traffic flow prediction results. Many situations can cause missing and abnormal traffic data, such as data anomalies caused by sensor failures and missing data during data transmission. Noise points are irregularly distributed in the data set, and the judgment of noise points is mainly based on the distribution of data before and after the noise points. If a point is significantly higher or lower than the data before and after, or the value is higher than the normal situation, we judge it as a noise point and remove it. After processing the noise points, it will produce discontinuity of time series, i.e. missing data. In addition, the data itself may miss some points of data because of equipment failure and other reasons, so it is significant for data repairing. The processing methods for missing data are divided into short-time missing and long-time missing. Short-time missing refers to missing data around 5 min. We directly use the last time point instead. Long-time missing refers to the absence of multiple consecutive time points, and we take the average value of the historical data for the same period as a substitute. Finally, the data are smoothed based on the use of the locally estimated scatterplot smoothing method. The processed data is smoother and more continuous than the raw data, which is consistent with the real situation and helps the neural network’s training, and improves the neural network’s prediction performance.

**Figure 2:**
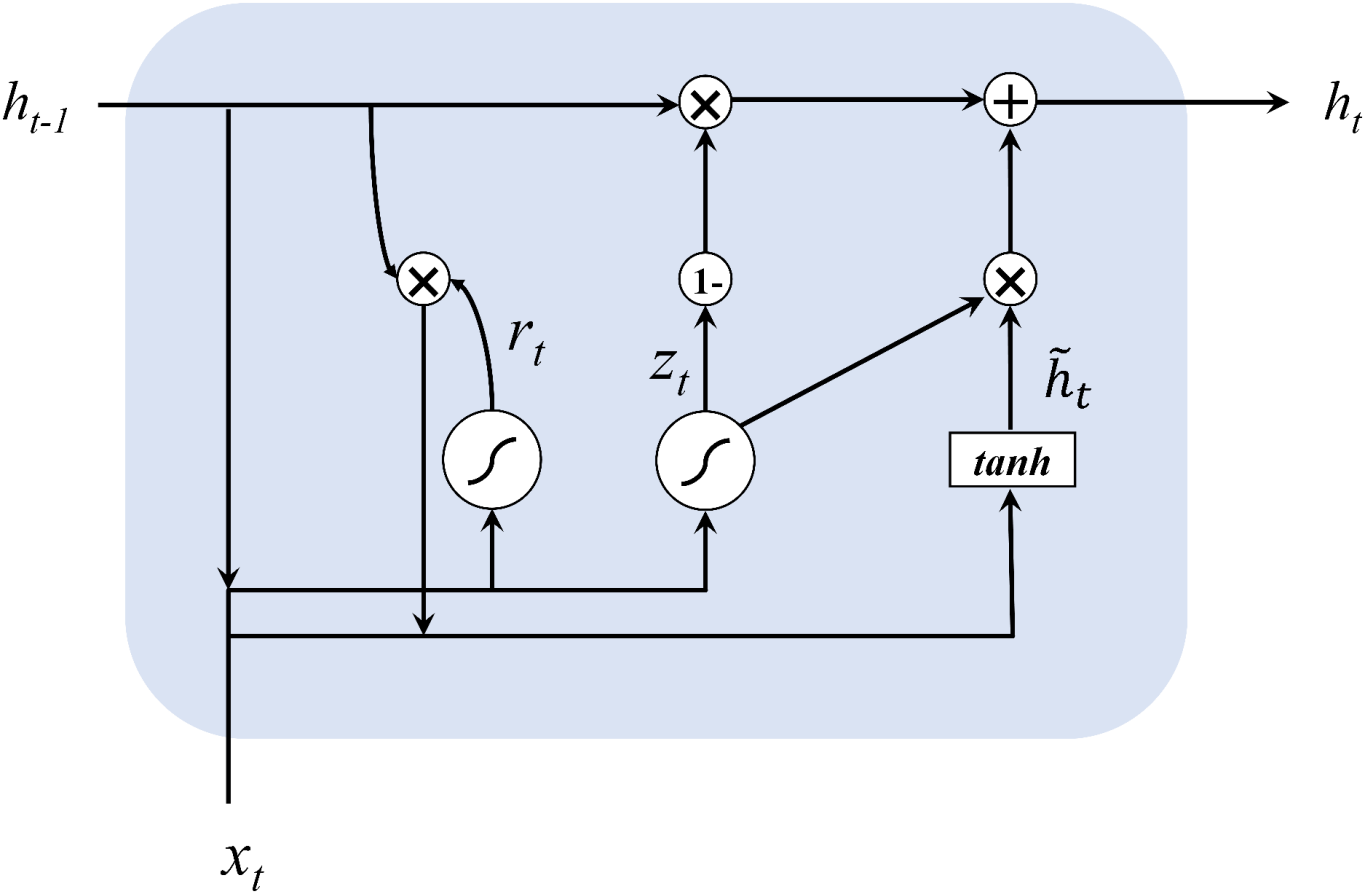
The structure of GRU networks.

This article uses neural network and time series methods to analyze dynamic traffic flows. In order to ensure real-time optimization, we first make a short-term prediction of the traffic flow, because accurate traffic flow forecasting is a key part of the overall framework. This article uses GRU neural network for online traffic flow prediction. The neural network can better describe the randomness and nonlinearity of traffic flow (Fu, Zhang & Li, 2016) and get more accurate prediction results compared to linear models such as ARIMA. GRU was proposed by Cho et al. (2014) and is very similar to LSTM, but is simpler to compute compared to LSTM. Figure 2 shows the structure of GRU neural network.

The input and output structure of GRU is similar to a typical recurrent neural network. The hidden state of memory cells is computed in the following formulas:

(1) }{}{z_t} = {\rm\sigma} ({W_z} \cdot [{h_{t - 1}},{x_t}])

(2) }{}{r_t} = {\rm\sigma} ({W_r} \cdot [{h_{t - 1}},{x_t}])

(3) }{}{\tilde h_t} = \tanh (W \cdot [{r_t} * {h_{t - 1}},{x_t}])

(4) }{}{h_t} = (1 - {z_t}) * {h_{t - 1}} + {z_t} * {\tilde h_t}

For the current node, the input values are the current input *x*^*t*^ and the hidden state *h*^t−1^ containing the information of the previous node, and the output values are the output *y*^*t*^ of the current node and the hidden state *h*^*t*^ containing the information of this node. GRU first obtains the gating states *z* and *r* by two input values, where *z* is the gate that controls the update and *r* is the gate that controls the reset. Then it calculates the candidate hidden layer *h^t^*, which represents the new information at the current moment, and controls the retention of the previous memory with *r*. Finally, both forgetting and remembering steps are performed simultaneously, using the update gate *z* to control the amount of information that needs to be forgotten from the previous moment’s hidden layer *h*^*t*−1^, and the amount of memory for the currently hidden layer information *h^t^*.

### Traffic flow analysis

Once the predicted values of the mainline and ramp flow are obtained, we measure the flux-correlation between flow-series, to find the relationship between mainline and ramps. In this article, we choose the cross-correlation algorithm (Paparrizos & Gravano, 2015) to measure the correlation, which is a similarity measure for time-lagged time series of traffic flow. The value of cross-correlation ranges between [−1, 1], while closing to 1, indicating a strong correlation and a strong negative correlation close to −1. For the time series *X* = (*x*_1_, *x*_2_, …, *x*_n_) and *Y* = (*y*_1_, *y*_2_, …, *y*_n_), the reciprocal method holds *Y* stationary so that *X* slides along *Y*. For each slide *s* of *X*, calculate their inner product as shown in the following equation.

(5) }{}X = (x_{\rm 1}, x_{\rm 2}, \ldots, x_{\rm n})

(6) \eqalignb{Y_s = \left\{\matrix {(\overbrace{0,\ldots ,0}^{|s|} , y_1, y_2,\ldots, Y_{n-s}) S \ge 0 \cr (y_{1-s},\ldots,y_{n-1},y_n, \underbrace{0,\ldots,0}_{|s|} )  S \lt 0}\right.}

For all possible slides *s*, the inner product CC (*X, Y*) is computed as the similarity between the two time series *X* and *Y*. The equation is shown below.

(7) }{}{\rm CC}(X,Y) = \left\{ {\matrix{ {\sum\limits_{i = 1}^{n - s} {{x_{s + i}} \cdot {y_i}} }  {s \ge 0} \cr {\sum\limits_{i = 1}^{n + s} {{x_i} \cdot {y_{i - s}}} }  {s < 0} \cr } } \right.

The cross-correlation is the maximum value of the inner product, which represents the similarity between *X* and *Y* under optimal phase sliding *s* conditions. Because under optimal sliding conditions, pattern-similar *X* and *Y* are exactly aligned, the internal product of both is maximal. Therefore, the Cross-correlation method overcomes the phase sliding problem and compares the shape similarity of two-time series. In practice, the normalized value of Cross-correlation is usually used to limit the range to be within [−1, 1], where 1 means strong correlation and −1 means that they are completely opposite. Besides, a positive NCC means that two series are in the same direction, while a negative NCC means that when one series tends to increase, the other tends to decrease and vise versa. The Eq. (8) defines NCC.

(8) }{}NCC(X,Y) = \mathop {\max }\limits_s (\displaystyle{{CC(X,Y)} \over {{{\left\| X \right\|}^2} \cdot {{\left\| Y \right\|}^2}}})

Generally, the distance between the bottleneck and ramp determines the weight matrix. In this article, we combine several factors to determine the weight matrix, including correlation, predicted ramp flow and distance. We measure the cross-correlation between the predicted flow-series of bottleneck and upstream ramps. If the value is greater than 0, it means that the ramps positively correlated with the bottleneck. The magnitude of the coefficient represents the degree of correlation. The value of correlation represents by NCC, together with the physical distance *d(i, j)* between the ramp and the bottleneck, and the predicted ramp traffic flow *q(j)* are taken as feature parameters of the weight matrix. If the number of correlations is less than 0, it means that the ramp flow is negatively correlated with the bottleneck flow, so the correlation has the opposite effect. Therefore, we only consider the physical distance and the predicted ramp flow as the feature parameters of the weight matrix.

As Eq. (9) shows, *w(i, j)* represents the weight of the *j*-th ramp for the *i*-th bottleneck. *d(i, j)* and *q(j)* are normalized parameters. \mu represents the correlation limitation, less than which means weak correlation between the time series, while a greater set shows strong correlation. This value is generally intermediate, in this Equation is 0.5. \rm\alpha, \rm\beta represents the weight of the temporal correlation coefficient, in the weak correlation condition, correlation shows light impact, while the strong correlation condition has a greater impact.

(9) }{}w(i,j) = \left\{ {\matrix{ {{{\rm\alpha}_{\rm 1}} \cdot d(i,j){\rm + }{{\rm\beta}_{\rm 1}} \cdot q(j)}  {{\rm NCC} \in [ - 1,0]} \cr {{{\rm\alpha}_{\rm 2}} \cdot {\rm NCC}(i,j) + {{\rm\beta}_{\rm 2}} \cdot d(i,j){\rm + (1} - {{\rm \alpha} _{\rm 2}} - {{\rm\beta}_{\rm 2}}{\rm )} \cdot q(j)}  {{\rm NCC} \in (0,{\rm\mu}]} \cr {{{\rm\alpha}_3} \cdot {\rm NCC}(i,j) + {{\rm\beta}_3} \cdot d(i,j){\rm + (1} - {{\rm\alpha}_3} - {{\rm\beta}_3}{\rm )} \cdot q(j)}  {{\rm NCC} \in ({\rm\mu} ,1]} \cr } } \right.

To explore the factors that are most relevant to the traffic states, (Yu et al., 2019) used the gray correlation analysis to measure the similarity between the flow, occupancy, density curves, and the speed curves. Grey correlation determines the degree of association between two factors based on how similar or dissimilar the trends are. It is often used to determine the relative strength of a project influenced by other factors. In this article, we compare the bottleneck speed curves with correlation value, the flow value of the on-ramps and the distance to optimize the parameters in Eq. (9).

For }{}Att{r_{i,j}} = ({a_{1,j}},{a_{2,j}},\ldots,{a_{k,j}}),{\rm }{a_{i,j}} \in \left\{ {\rm ncc,inflow,distance} \right\}, we can obtain the degree of association }{}GR{C_{i,j}} = (gr{c_{1,j}},gr{c_{2,j}},\ldots,gr{c_{k,j}}),\sum\nolimits_{i = 1}^k {gr{c_{i,j}} = 1}, and then the influence degree of ramp inflow and downstream speed. The influence degree shows how much influence will the factor affects downstream speed as Eq. (10), where the *rs* means road section number and *k* represents the factor number.

(10) }{}{\rm InfluenceDegree_{\rm inflow}} = \displaystyle{1 \over {rs}}\sum\limits_{i = 1}^k {\sum\limits_{j = 1}^{rs} {gr{c_{i,j}}} }

Equation (11) calculates the final weight matrix. Where *m* is the number of bottlenecks, this weighting matrix will be used in signal coordination optimization to assign the excess traffic demand, in order to ensure the rationality of the traffic reduction at each ramp.

(11) \eqalignb{ W_{ij}= \left|\matrix{w(1,1)   \cdots   w(1,m)\cr  \ddots  \cdots  \ddots \cr 0  \vdots  w(i,j)  \vdots  \vdots\cr  0  \cdots  \ddots  \cr 0   0   w(n,m)}}\right|}

### Coordinated signal optimization

The coordinated signal optimization method proposed in this article implements a dynamic weight matrix between the ramp and mainline. The application of this matrix in the bottleneck algorithm is mainly reflected in the calculation of the coordinated metering rate. The bottleneck algorithm first determines whether the road segment downstream of the ramp is a bottleneck based on the real-time occupancy or density of the road segment. When there are no bottlenecks in the multi-ramp area, the local metering rate is applied to each on-ramp. When a bottleneck generates in a road segment, the excess traffic demand for each bottleneck generated needs to be allocated to each ramp. The more restrictive value of the coordinated and local metering rates is taken, and the final ramp metering rate is obtained by considering the ramp queue length limitation.

**Figure 3:**
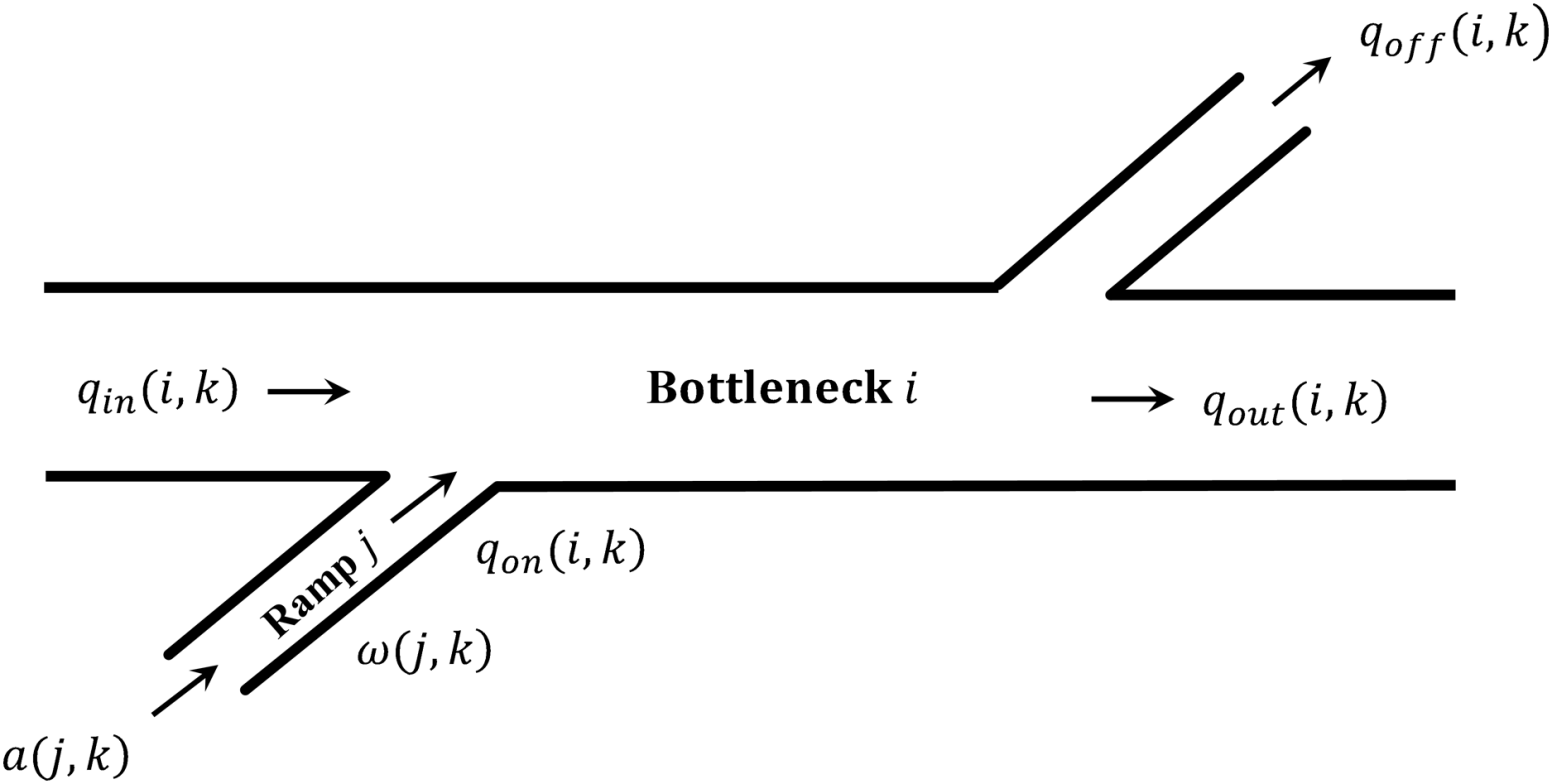
Bottleneck section.

Figure 3 shows a segment of multi-ramp road section. Initially, the local metering rate }{}{r_L} of each ramp at *k* time is calculated according to Eq. (12). The *K* represents metering parameter, and \hat {\rm\rho} is the critical density of ramp downstream road section, }{}{\rm\rho} (k - 1) is average density of downstream road section at last time step.

(12) }{}{r_L}(j,k + 1) = {r_L}(j,k) + K \cdot [\hat {\rm\rho} - {\rm\rho} (k)]

Defining the difference between the entering flow (including the upstream inflow from the mainline and the flow entering the entrance ramp), and exiting flow (including the downstream outflow from the mainline and the flow exiting the exit ramp) as the excess demand for that segment, see Eq. (13).

(13) \Delta d(i,k + 1) = {q_{\rm in}}(i,k) + {q_{\rm on}}(i,k) - {q_{\rm out}}(i,k) - {q_{\rm off}}(i,k)

If the average density in a control cycle is higher than the threshold (Eq. (14)); at the same time the excess demand is more than zero (Eq. (15)), there is a risk of breakdown, defining such a segment as a bottleneck.

(14) }{}{\rm\rho} \left( {i,k} \right) > {{\rm\rho} _{\rm threshold}}\left( i \right)

(15) \Delta d(i,k + 1) > 0

It is necessary to calculate the coordinated metering rate to eliminate bottlenecks, and the coordinated metering rate is calculated as shown in the Algorithm 1.

**Algorithm 1:** Coordinate metering rate calculation.

**Input:** Road section i; weight matrix *W*_*ji*_
**Output:** Coordinate metering rate *r_C_*
1 **for** *i in road section* **do**
2 **if** *density* > *threshold and Excess demand* > 0 **then**
3 *q_reduction_* (*i*, *k* + 1) = Δ*d* (*i*, *k* + 1);
4 *q_reduction_* (*i*, *j*, *k* + 1) = *q_reduction_* (*i*, *k* + 1) ⋅*W_ji_*;
5 **else** *i* = *i* + 1
6 *r_C_* (*j*, *k* + 1) = *r* (*j*, *k*) − max*_i_* [*q_reduction_* (*i*, *j*, *k* + 1)];
7 return *r_C_*

Every bottleneck needs to reduce the excess demand \Delta d(i,k + 1), when \Delta d(i,k + 1) < 0, the bottleneck is eliminating, }{}{q_{\rm reduction}}(i,k + 1) = 0. Eq. (17) shows the volume reduction of every ramp, }{}{W_{ji}} is weight matrix. The max volume reduction is selected to calculate the coordinate metering rate in Eq. (18), based on metering rate *r*(*j*, *k*) last time step.

(16) }{}{q_{\rm reduction}}(i,k + 1) = \Delta d(i,k + 1)

(17) }{}{q_{\rm reduction}}(i,j,k + 1) = {q_{\rm reduction}}(i,k + 1) \cdot {W_{ji}}

(18) }{}{r_C}(j,k + 1) = r(j,k) - \mathop {\max }\limits_i [{q_{\rm reduction}}(i,j,k + 1)]

The system metering rate adopts the more restrictive of coordinate metering rate and local metering rate as Eq. (19) shows.

(19) }{}{r}^{\prime}(j,k + 1) = \min [{r_L}(j,k + 1),{r_C}(j,k + 1)]

If the ramp metering rate approaches zero, it will cause a ramp spillback, thus affect surface traffic. Therefore, the metering rate needs to be constrained based on the queue length of the ramp. In Eq. (20), }{}a(j,k) is the arrival rate of vehicles entering the entrance ramp, \hat {\rm\omega}(j) is the capacity of the *j*-th ramp, }{}{\rm\omega}(j,k) is the queue length of the *j*-th on-ramp at the previous time step, and T refers to a time step. The metering rate can be obtained by Eq. (21).

(20) }{}{r_{\rm queue}}(j,k + 1) = a(j,k) - \displaystyle{1 \over T}[\hat {\rm\omega} (j) - {\rm\omega}(j,k)]

(21) }{}r(j,k + 1) = \max [{r}^{\prime}(j,k + 1),{r_{\rm queue}}(j,k + 1)]

The green duration is calculated based on the metering rate and sent to each signaler in Eq. (22). Where *C*(*j*) indicates the signal cycle duration and *r*_s_(*j*) indicates the saturation flow rate.

(22) }{}g(j,k + 1) = \displaystyle{{r(j,k + 1)} \over {{r_s}(j)}} \times C(j)

## Experiment

### Traffic Flow Prediction

**Figure 4:**
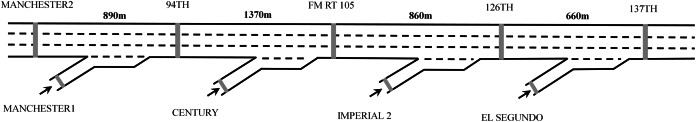
Selected detectors and deployment locations.

The dataset used in this article is collected from the California PEMS platform, which provides 5-min intervals of freeway mainline loop detector data (both speed and flow) and on-ramp passing data. We select the flow data for the northbound upstream mainline and on-ramp of the I110 freeway from 1 January 2020 to 26 February 2020, which has four on-ramps with a total length of approximately 3.8 KM. The detector deployment locations show in Fig. 4.

**Figure 5:**
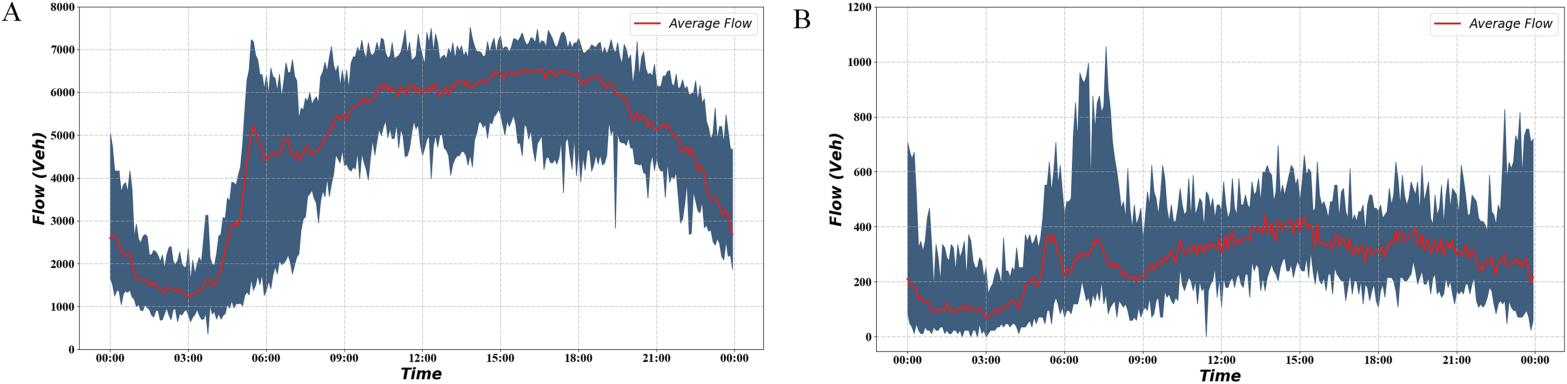
Traffic volume during 2 months. (A) Freeway mainline traffic volume during 2 months. (B) Freeway ramp traffic volume during 2 months.

Combined with the local traffic flow characteristics, it can be found that the traffic flow is at its peak during 15:00–18:00, which is suitable for applying ramp control. Ramp control is not sufficient when the traffic flow is low, smooth traffic flow can be achieved using no control or simple demand capacity control (Papageorgiou & Kotsialos, 2002). We plotted the average traffic flow curve of the bottleneck road section over 2 months in Fig. 5.

**Figure 6:**
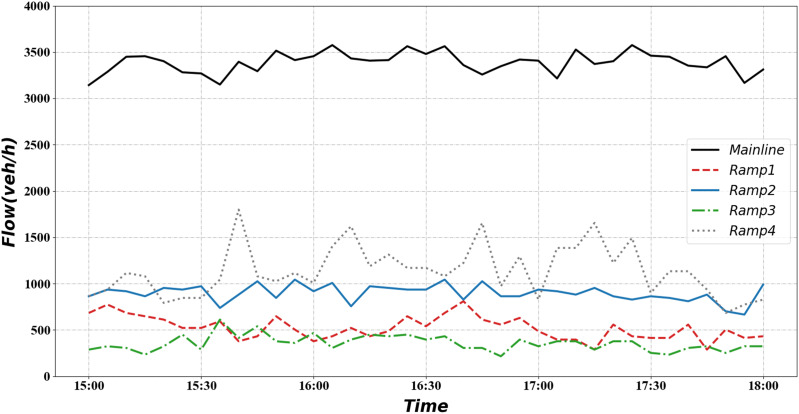
Mainline and ramp flow demand.

The red curve in the figure indicates the average traffic flow on the road section during the 2 months. The blue part indicates the interval formed by the maximum and minimum traffic volumes. It can be seen from the graph that the traffic flow starts to increase at 6:00 and stays at a high level for a long time afterward. The interval we selected is the time segment with relatively high traffic volume, which helps verify the effectiveness of the signal optimization scheme. Based on the above findings, flow data from 15:00 to 18:00 on 13 February 2019 upstream of the mainline and the four ramps were selected as inputs, as shown in Fig. 6.

In this article, the GRU neural network is used for traffic flow prediction. The best lag time can minimize the prediction error of the model, and we use the maximum lag time of 60 min. It is more reasonable to use 12 historical values to predict the latter value, and fewer historical values lead to more deviations in the prediction results. More historical values lead to long computation time and are prone to overfitting. In addition to the lag time, we also need to choose the best parameters for the deep neural network model. We use a two-layer GRU neural network with 64 hidden cells in each layer, a batch size of 256, and a max epoch of 600. The training set uses forty-eight days of data (84.2% of the total number of days), the test set uses the remaining nine days of data (15.8%).

We compare the GRU neural network with two other methods, SAEs and LSTMs, respectively. We use the rooted mean squared error (RMSE) and mean absolute percentage error (MAPE) as the metric to evaluate these methods. When the predicted value exactly matches the true value, RMSE is equal to 0, indicating a perfect model. RMSE can be calculated as follows:

(23) }{}{\rm RMSE} = \sqrt {\displaystyle{1 \over m}\sum\limits_{i = 1}^m {{{({y_i} - {{\hat y}_i})}^2}} }

where }{}{y_i} is the ground truth and }{}{\hat y_i} is the prediction value, *m* represents the total number of predictions.

Mean absolute percentage error of 0% indicates a perfect model, while a MAPE greater than 100% indicates a flawed model. MAPE can be calculate as follows:

(24) }{}{\rm MAPE} = \displaystyle{{100\% } \over m}\sum\limits_{i = 1}^m {\left| {\displaystyle{{{{\hat y}_i} - {y_i}} \over {{y_i}}}} \right|}

where }{}{y_i} is the ground truth and }{}{\hat y_i} is the prediction value, *m* represents the total number of predictions.

The Table 2 shows the prediction results of various methods. It can be seen that in the RMSE, both LSTM and GRU are much better than SAEs. There is little difference between LSTM and GRU. In the MAPE column, GRU performs the optimal prediction effect.

**Table 2:** The performance comparisons.

Method	RMSE	MAPE (%)
*SAEs*	22.18	8.75
*LSTM*	14.95	4.21
*GRU*	15.21	4.14

**Figure 7:**
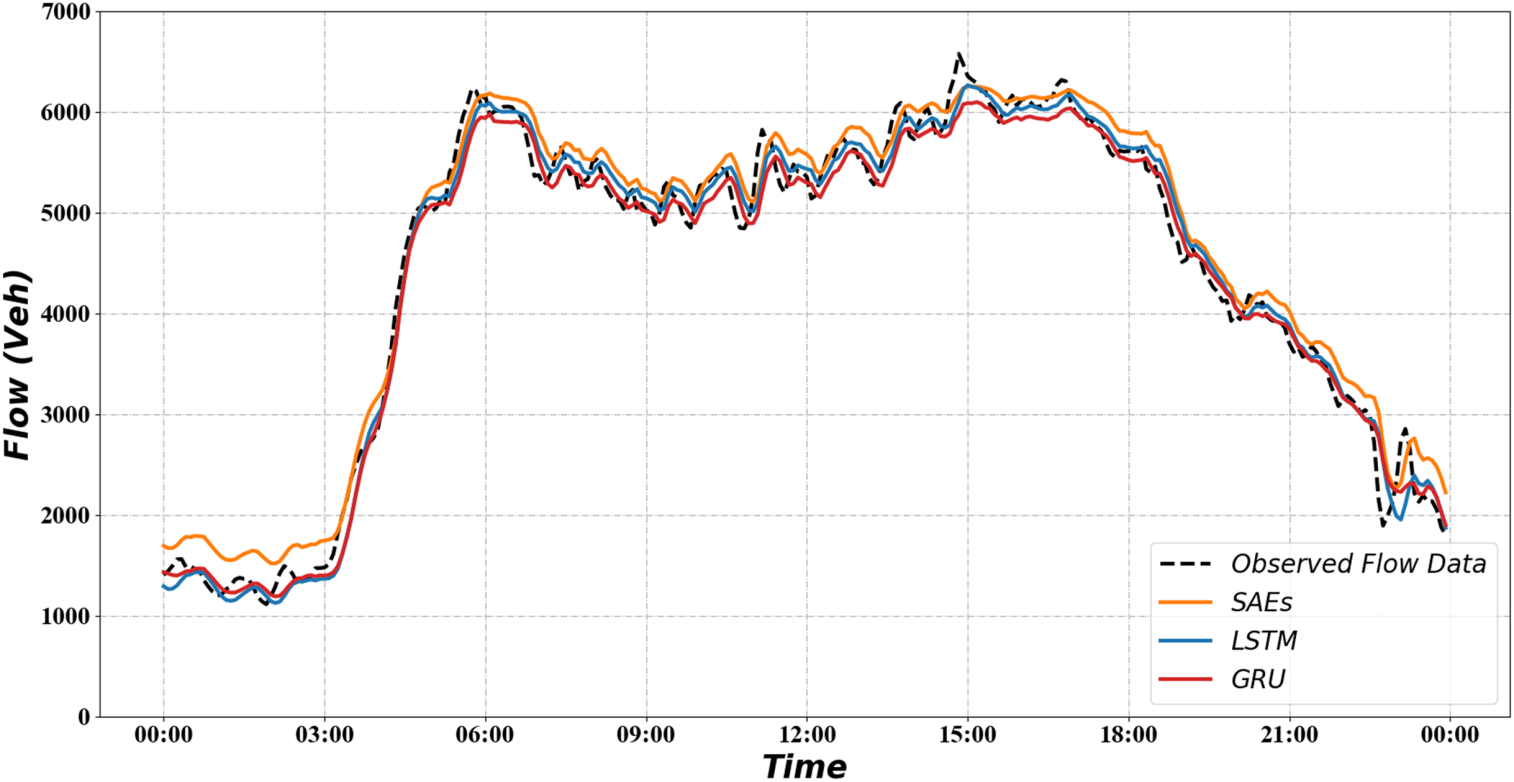
Result of traffic flow prediction.

Figure 7 shows how the predicted value compares with the observed value and other methods. It can be seen from the figure that all curves fit the observed data. During the low-flow phase, the SAEs were predicted to be substantially higher than the true values. And the LSTM and GRU are much closer to the true values.

### SUMO platform

**Figure 8:**
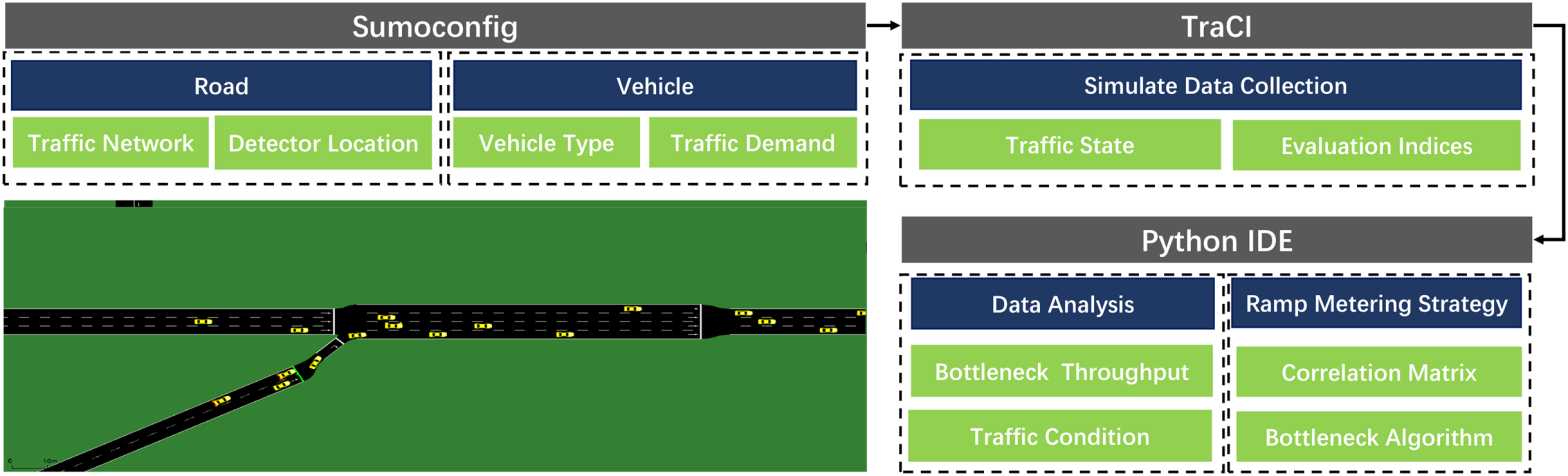
SUMO simulation framework.

Simulation of Urban Mobility (Lopez et al., 2018; Krajzewicz et al., 2012) used in this article is a free and open-source traffic system simulation software compared with the above microsimulation platforms. It can realize microscopic control of traffic flow. Each vehicle’s route on the specific road can be planned individually. Furthermore, it has a mature code control module, which makes data analysis more convenient. The entire SUMO-based simulation framework shows in Fig. 8.

We model multi-ramp section in SUMO, and entering the traffic flow data during the morning peak period. We use the IDM model as car following model. The IDM model (Treiber, Hennecke & Helbing, 2000) is by far the most complete and simple accident-free theoretical heeling model, which belongs to the class of expectation measures. The IDM model is described in Eqs. (25) and (26).

(25) }{}{{v}^{\prime}_{\rm\alpha}} = a\left[ {1 - {{\left( {\displaystyle{{{v_{\rm\alpha}}} \over {{v_0}}}} \right)}^{\rm\delta}} - {{\left( {\displaystyle{{{s^ * }\left( {{v_{\rm\alpha}},\Delta{v_{\rm\alpha}}} \right)} \over {{s_{\rm\alpha}}}}} \right)}^2}} \right]

(26) }{}{s^ * }\left( {v,\Delta v} \right) = {s_0} + {s_1}\sqrt {\displaystyle{v \over {{v_0}}}} + {T^{\rm\alpha}}v + \displaystyle{{v\Delta v} \over {2\sqrt {ab} }}

The vehicle acceleration }{}{{v}^{\prime}_{\rm\alpha} } can be obtained by minimum vehicle distance }{}{s^ * }\left( {v,\Delta v} \right), velocity, vehicle clearance from the vehicle in front }{}{s_\alpha } and expected velocity }{}{v_0}. In Eq. (26), T represents safety time step, }{}ab represent the max acceleration and the expected deceleration ability of vehicles. \Delta v is speed difference with the car in front, }{}{s_0} and }{}{s_1} are distance with congested traffic. For model parameter calibrations, see Table 3 below.

**Table 3:** Model parameters of IDM.

Attribute	Value	Description
*v*_0_	18.87 m/s	Expected velocity of vehicles
*a*	2.6 m/s	The max acceleration ability of vehicles
*b*	4.5 m/s	The expected deceleration ability of vehicles
*s*_0_, *s*_1_	2.5 m	Distance with congested traffic
*T*	2 s	Safety time step
δ	4	Acceleration index
*l*	5 m	Vehicle length

Besides, the channel change model is also very critical. The vehicle lane change model we have chosen is LC2013 (Erdmann, 2015).

In conjunction with the application in the related literature (Papamichail et al., 2010a), the common evaluation metrics are Average Travel Time (ATT), which indicates the travel time of mainline vehicles; Average Waiting Time (AWT), which indicates the waiting time of ramp vehicles. Besides, the density and speed of the bottleneck locations are selected as evaluation metrics, which can reflect the ability to alleviate congestion at the bottleneck locations.

## Results

This article uses SUMO simulation software to perform experiments on four on-ramp scenarios. The correlation between individual ramps and bottlenecks can be obtained by the method above, leading to the weight matrix. We have used various methods for simulation.

**No signal method:** ramp signal is all green. In this case, the on-ramp traffic can enter the freeway without obstruction. This is used as a control experiment to demonstrate the effect of signal optimization.

**Distance-based method:** only the spatial distance between the ramp and the bottleneck is used as a weighting factor for traffic assignment. Such an approach would overweight the ramp closest to the bottleneck and assign less weight to the other ramps upstream. Such an approach has an absolute static nature.

**Distance-flow-based method:** use spatial distance and the on-ramp flow as the assigned weight factors. Such an approach considers the contribution of the on-ramp flow to the bottleneck congestion compared to considering only the spatial distance. However, the on-ramp flow may flow at other off-ramps and is not necessarily the root cause of congestion.

**Traffic states-based method:** use spatial distance, ramp flow, and dynamic traffic flow correlation as the assigned weighting factors. Such a method adds the traffic flow correlation between ramps and bottlenecks to the above method. It better traces the source of congestion and is dynamic and predictable.

Table 4 shows the results of the weights matrix generated from different factors. Our correlation measurement of traffic flow-series reveals that the closest on-ramp upstream may not significantly impact the bottleneck. A particular ramp further upstream may have a greater impact on the bottleneck because of the faster flow growth trend.

**Table 4:** Weight matrix based on different factors.

		Bottleneck 1	Bottleneck 2	Bottleneck 3	Bottleneck 4
Distance based	Ramp 1	1.00	0.04	0.02	0.02
	Ramp 2	0.00	0.96	0.05	0.03
	Ramp 3	0.00	0.00	0.93	0.06
	Ramp 4	0.00	0.00	0.00	0.89
Distance and flow based	Ramp 1	1.00	0.20	0.16	0.10
	Ramp 2	0.00	0.80	0.28	0.17
	Ramp 3	0.00	0.00	0.56	0.09
	Ramp 4	0.00	0.00	0.00	0.64
Traffic states based	Ramp 1	1.00	0.20	0.07	0.04
	Ramp 2	0.00	0.80	0.18	0.07
	Ramp 3	0.00	0.00	0.76	0.25
	Ramp 4	0.00	0.00	0.00	0.64

**Figure 9:**
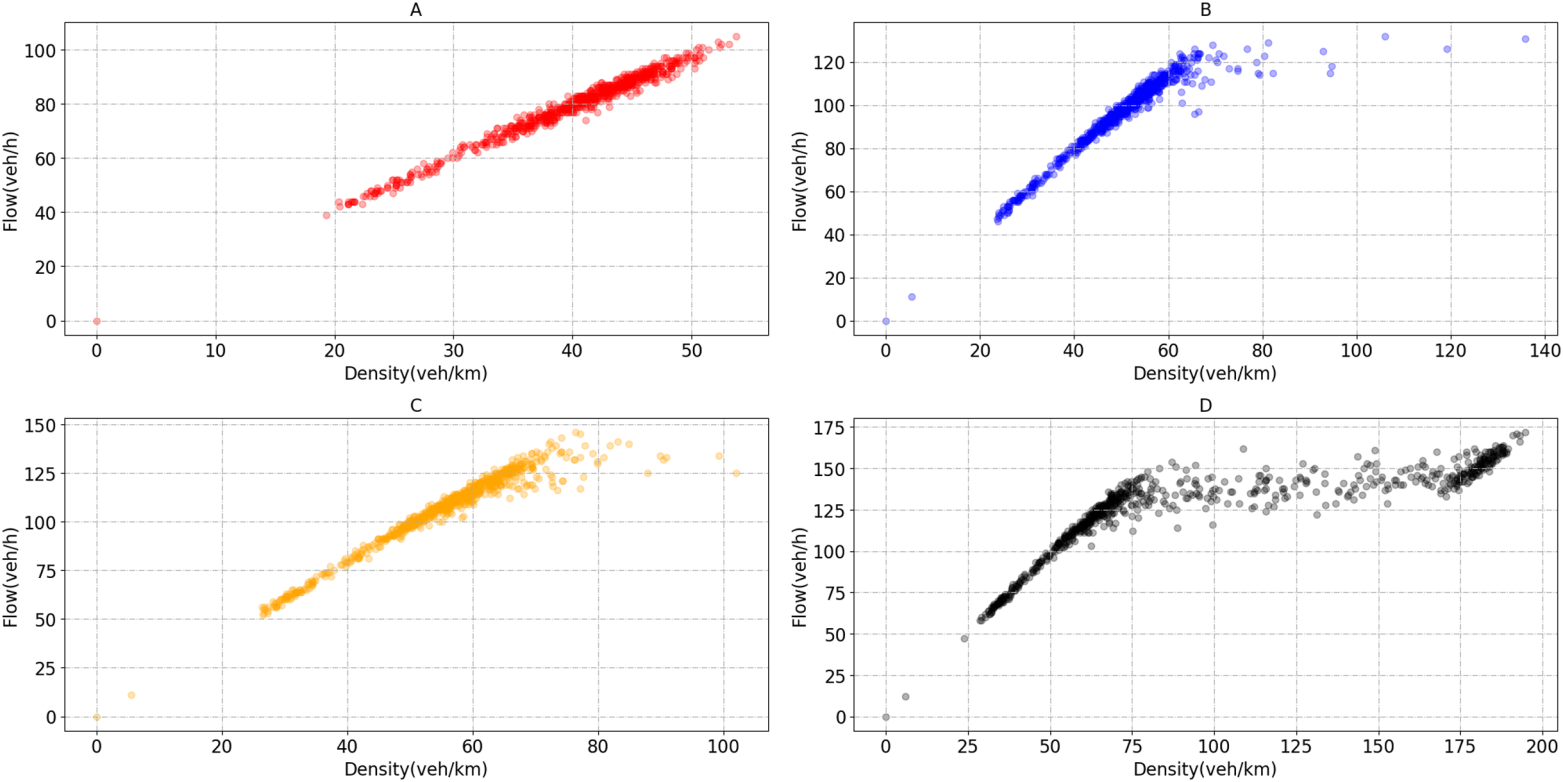
Flow-density scatter plot of bottleneck section. (A) Flow and density of bottleneck1. (B) Flow and density of bottleneck2. (C) Flow and density of bottleneck3. (D) Flow and density of bottleneck4.

Before implementing the signal optimization algorithm, each bottleneck road section's critical density needs to be obtained. As the traffic flow is sensitive to the critical density changes, the traffic state can be divided near the critical point. The four bottleneck sections’ critical densities are 60, 67, 75 and 90 veh/km, as shown in Fig. 9.

We test the performance of the algorithm under normal and heavy demands. There are five lanes and two lanes in the mainline and ramp, separately. So, we use 9,000 pcu/h as mainline saturated flow rate and 3,600 pcu/h as ramp saturated flow rate (Niittymäki & Pursula, 1997). According to the Fig. 5A, we can observe that between 15:00 and 18:00, the road section’s average traffic flow is about 6,500 pcu/h, which is about 72.2% of the saturated flow rate. Moreover, the maximum value is approximately 7,300 pcu/h, about 81.1% of the saturation flow rate. In the simulation process, we found that using real mainline demand can cause a congestion situation. Instead of increasing the mainline traffic, we set up a scenario of increasing ramp traffic to simulate heavy ramp traffic and evaluate the signal optimization scheme. As shown in the Fig. 5B, the average traffic flow on the ramp between 15:00 and 18:00 is about 300 pcu/h. This value is far below the saturated flow rate of 3,600 pcu/h. Therefore, we added a heavy demand to simulate the busy condition of the ramp. The traffic flow under heavy demand is about 450–500 pcu/h, accounting for about 12.5–13.8% of the saturation flow rate.

The normal demand represents releasing vehicles at 66.7% of the mainline saturated traffic demand, 8.3% of the ramp saturated traffic demand. The heavy demand releases vehicles at 66.7% of the mainline saturated traffic demand, 13.8% of the ramp saturated traffic demand. Table 5 below shows the performances of each algorithm. It shows that the mainline ATT improvement using the ramp signal optimization method is limited in normal demand. In contrast, it has a larger improvement in the ramp AWT.

**Table 5:** Performance under different demand.

Demand	Method	Mainline travel time (s)	Ramp waiting time (s)	Bottleneck density (veh/km)	Bottleneck velocity (km/h)
Normal demand	No signal	269.55	7.42	90.06	23.05
	Distance based	264.35 (−1.9%)	8.86 (+19.4%)	85.69 (−4.9%)	23.92 (+3.8%)
	Distance and flow based	264.06 (−2.0%)	8.95 (+20.6%)	84.02 (−6.7%)	23.89 (+3.6%)
	Traffic states based	263.47 (−2.2%)	8.93 (+20.3%)	83.62 (−7.1%)	24.26 (+5.2%)
Heavy demand	No signal	285.02	11.65	118.20	22.45
	Distance based	272.21 (−4.5%)	13.72 (+17.8%)	107.90 (−8.7%)	23.27 (+3.6%)
	Distance and flow based	270.15 (−5.2%)	12.92 (+10.9%)	105.03 (−11.2%)	23.44 (+4.4%)
	Traffic states based	269.25 (−5.5%)	12.67 (+8.8%)	102.82 (−13.0%)	23.46 (+4.5%)

Because when traffic demand is low on the mainline, the on-ramp merges into the mainline, and the ramp signal reduces movement efficiency. In terms of movement through the bottleneck, the traffic-state-based method reduces bottlenecks’ density to a greater extent (7.1%) than the other approaches (4.9% and 6.7%). Compared to other methods, our method has a smaller gap in speed through the bottleneck, but the improvement is relatively significant (5.2%). The heavy demand scenario simulates over-congestion during peak periods. The signal optimization is more pronounced than the no signal scenario, with 4.5%, 5.2% and 5.5% improvements in mainline ATT, respectively. However, a smaller increase in ramp AWT compared to normal demand. The approach that considers traffic states effectively reduces the bottleneck’s density under heavy demand, reducing it by an average of 15.38 veh/km (13%) and increasing 4.5% of the average speed of vehicles passing through the bottleneck.

**Figure 10:**
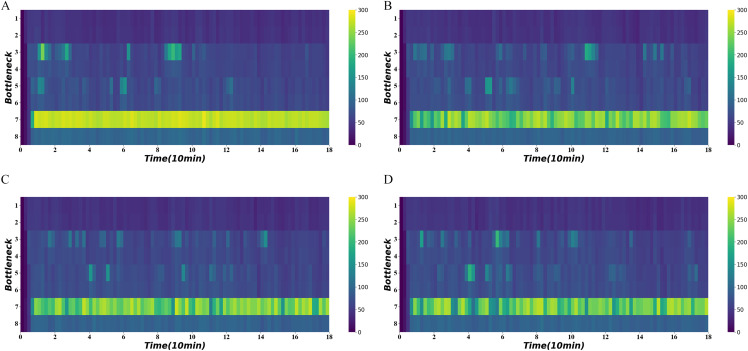
Density heatmap on normal demand. (A) No signal. (B) Distance-based method. (C) Distance-flow-based method. (D) Traffic-state-based method.

Figure 10 shows the average density of the four bottlenecks under normal demand using different signal optimization algorithms. It demonstrates persistent congestion in the seventh road section, for which no control is often not possible. In contrast, the use of signal optimization can significantly reduce congestion. The distance-flow-based method and traffic-state-based method have a better performance on the congestion reduction. The traffic-state-based method keeps density to a much lower range in 40–60 min.

**Figure 11:**
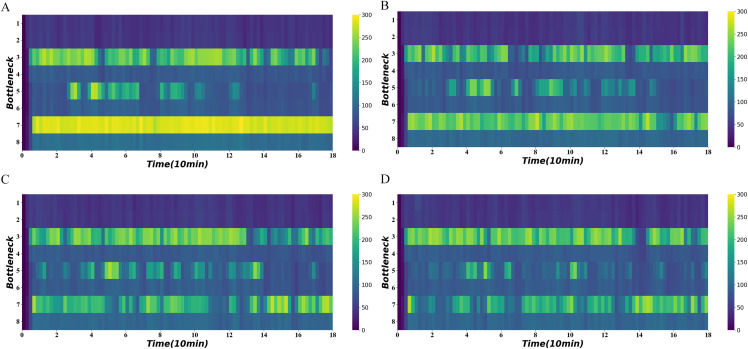
Density heatmap on heavy demand. (A) No signal. (B) Distance-based method. (C) Distance-flow-based method. (D) Traffic-state-based method.

Figure 11 shows the average density of the four bottlenecks under heavy demand using different algorithms. It shows that the third and fifth road sections also experience intermittent congestion in addition to the seventh road section. The traffic-state-based method effectively relieves congestion in the fifth and seventh road segments and maintains a lower density at 20–60 and 120–140 min comparing to the others.

**Figure 12:**
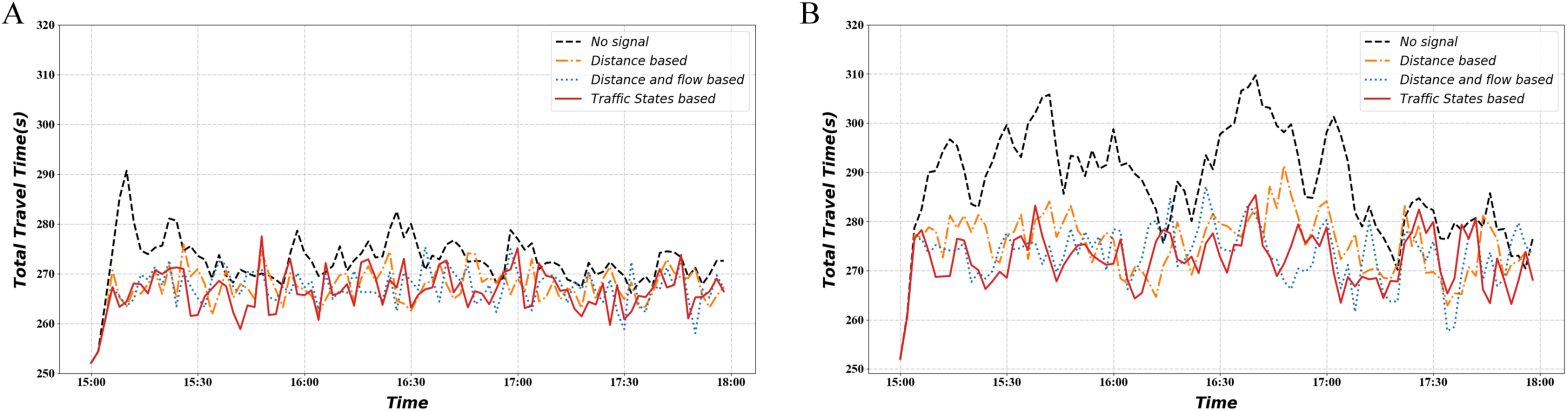
Total travel time. (A) Normal demand situation. (B) Heavy demand situation.

Figure 12 shows the variation in mainline AWT under different demand conditions. It illustrates that the signal optimization method keeps the mainline ATT relatively low regardless of the demand level. The traffic-state-based method exhibits lower ATT at more moments, which means smoother vehicle movement.

## Conclusions

To address expressway congestion, we propose a coordinated signal optimization framework based on dynamic traffic states. In this article, the GRU neural network was used to predict the traffic flow. Cross-correlation obtains the dynamic correlation between the bottleneck and ramp traffic flow. Our framework considers both static road property and dynamic traffic state to achieve signal optimization compared with previous research. The performance of our algorithm was verified on the SUMO simulation platform. The results show that our approach can reduce the mainline bottleneck density by 7.1% and 13.0% under normal and heavy demand, separately. We also found that it is a more reasonable option in the lower traffic demand scenario without using a signal. Moreover, in congested scenarios, the use of signal optimization can reduce the density of the mainline.

In future work, we will work on signal optimization solutions for the broad ramp area. Using detailed traffic trajectory data to analyze congestion’s spatial and temporal characteristics, we will develop a coordinated signal optimization program.

## Additional Information and Declarations

### Data Availability

The following information was supplied regarding data availability: Data and code are available at GitHub: https://github.com/wendyshu43/SignalOpt.
